# Demethylation of H3K9 and H3K27 Contributes to the Tubular Renal Damage Triggered by Endoplasmic Reticulum Stress

**DOI:** 10.3390/antiox11071355

**Published:** 2022-07-12

**Authors:** Paula Diaz-Bulnes, Maria Laura Saiz, Viviana Corte-Iglesias, Raúl R Rodrigues-Diez, Aida Bernardo Florez, Cristian Ruiz Bernet, Cristina Martin Martin, Marta Ruiz-Ortega, Beatriz Suarez-Alvarez, Carlos López-Larrea

**Affiliations:** 1 Translational Immunology, Instituto de Investigación Sanitaria del Principado de Asturias ISPA, 33011 Oviedo, Spain; uo202451@uniovi.es (P.D.-B.); mlsa.inmuno@ispasturias.es (M.L.S.); vivi.inmuno@ispasturias.es (V.C.-I.); rrodriguez@fjd.es (R.R.R.-D.); uo252369@uniovi.es (A.B.F.); uo258121@uniovi.es (C.R.B.); cmartinsorting@finba.es (C.M.M.); inmuno@hca.es (C.L.-L.); 2 RICORS2040 (Kidney Disease), Instituto de Salud Carlos III, 28029 Madrid, Spain; marta.ruiz.ortega@uam.es; 3 Cellular Biology in Renal Diseases Laboratory, IIS-Fundación Jiménez Díaz, Universidad Autónoma, 28040 Madrid, Spain; 4 Servicio de Inmunología, Hospital Universitario Central de Asturias, 33011 Oviedo, Spain

**Keywords:** ER stress, epigenetic modifications, histone methylation, UPR

## Abstract

Loss of protein homeostasis (proteostasis) in the endoplasmic reticulum (ER) activates the unfolded protein response (UPR), restoring correct protein folding. Sustained ER stress exacerbates activation of the major UPR branches (IRE1α/XBP1, PERK/ATF4, ATF6), inducing expression of numerous genes involved in inflammation, cell death, autophagy, and oxidative stress. We investigated whether epigenetic dynamics mediated by histone H3K9 and H3K27 methylation might help to reduce or inhibit the exacerbated and maladaptive UPR triggered in tubular epithelial cells. Epigenetic treatments, specific silencing, and chromatin immunoprecipitation assays were performed in human proximal tubular cells subjected to ER stress. Pharmacological blockage of KDM4C and JMJD3 histone demethylases with SD-70 and GSKJ4, respectively, enhanced trimethylation of H3K9 and H3K27 in the *ATF4* and *XBP1* genes, inhibiting their expression and that of downstream genes. Conversely, specific G9a and EZH2 knockdown revealed increases in ATF4 and XBP1 expression. This is a consequence of the reduced recruitment of G9a and EZH2 histone methylases, diminished H3K9me3 and H3K27me3 levels, and enhanced histone acetylation at the *ATF4* and *XBP1* promoter region. G9a and EZH2 cooperate to maintain the repressive chromatin structure in both UPR-induced genes, ATF4 and XBP1. Therefore, preserving histone H3K9 and H3K27 methylation could ameliorate the ER stress, and consequently the oxidative stress and the triggered pathological processes that aggravate renal damage.

## 1. Introduction

**Figure 1:**
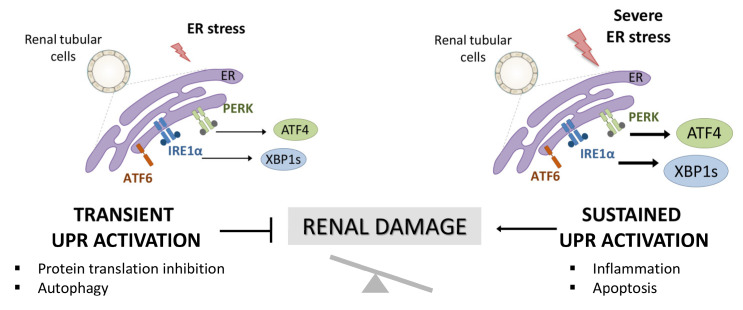
Activation of UPR in renal tubular cells and involvement in renal damage. Accumulation of unfolded proteins in the ER triggers the activation of the UPR pathway aimed at resolving the damage (transient UPR activation or adaptive UPR). However, when this UPR activation is sustained over time (maladaptive UPR), PERK/ATF4 and IRE1α/XBP1 UPR branches initiate pathological processes, such as exacerbated inflammation and apoptosis that aggravate the renal damage.

The endoplasmic reticulum (ER) is the central site of protein synthesis and transport, protein folding, lipid and steroid synthesis, and calcium storage. Alterations in the functions of the ER can result in the abnormal accumulation of unfolded protein, a cellular condition referred to as ER stress. To maintain the protein folding and ER functions, cells activate the unfolded protein response (UPR), a network of signal pathways that restore protein homeostasis (proteostasis) (Figure 1) [1]. The UPR pathway sets up an adaptive mechanism to maintain an optimal rate of protein production. However, when the pathogenic stimuli are too severe or sustained, protein homeostasis cannot be rectified and maladaptive mechanisms are triggered that lead to cell death by apoptosis and inflammation (Figure 1) [2].

Under physiological conditions, the three main ER stress sensors, IRE1α (inositol-requiring protein 1), PERK (protein kinase-like endoplasmic reticulum kinase), and ATF6 (activating transcription factor 6) are inactivated through binding to the chaperone BIP, also known as GRP78 (glucose-regulated protein 78), in the ER lumen. However, when the cells undergo ER stress, GRP78 binds to misfolded proteins, releasing these sensors and allowing their activation [3]. These sensors induce, through transcriptional and non-transcriptional processes, the upregulation of a subset of genes mediated by the XBP1, ATF4, and ATF6 transcription factors and the activation of diverse mechanisms aimed to minimize the protein folding load in the ER.

The disruption of the ER proteostasis and UPR activation affects the development of a broad range of kidney-related diseases such as glomerulosclerosis, glomerulonephritis, ischemia, diabetic nephropathy, nephrotoxicity, and chronic kidney disease (CKD) [4,5,6]. Therefore, inhibition of maladaptive UPR signaling has been proposed as a therapeutic goal to prevent or attenuate kidney disease progression. Most of the compounds assayed until now, are chaperones that restore ER-folding capacity. Treatment with 4-PBA (4-phenylbutyric) and TUDCA (taurine-conjugated ursodeoxycholic acid) lead to a decrease in the PERK/ATF4 and IRE1α/XBP1s signaling that ameliorates insulin resistance, apoptosis, inflammation, and fibrosis in in vivo diabetic nephropathy models [7,8,9]. In the renal damage induced by I/R, TUDCA administration exerts a nephroprotective effect by blocking GPR78 and CHOP expression and, consequently, cellular apoptosis, whilst 4-PBA attenuates the increased expression of pro-fibrotic genes [10,11,12]. Additionally, the inhibition of ER stress activation blocks the apoptosis induced by renal I/R injury through the prevention of FoxO4-dependent ROS generation [13]. However, the potential involvement of modulators specific to the downstream signaling pathways has been less thoroughly explored.

Here, we focus on the study of epigenetic modulators responsible for altering chromatin structure and, consequently, gene transcription. Previous studies have identified the blockage of epigenetic enzymes as a promising target for ameliorating renal damage [14,15,16]. Acetylation and methylation of lysine residues in histones are two of the key epigenetic modifications in regulating the transcription of genes related to inflammation, oxidative stress, fibrosis, and apoptosis, all of which are a consequence of maladaptive ER stress. Changes in the methylation levels of the H3K9 and H3K27 residues mediated by their respective histone methyltransferase (HMT), G9a and EZH2, or histone demethylase (HDMT), KDM4C and JMJD3, respectively, have been widely analyzed in the context of the development and progression of kidney disease [17,18,19,20]. However, their role in the ER stress triggered in tubular cells is yet to be elucidated. In this study, we explored the epigenetic dynamics mediated by the H3K9 and H3K27 methylation in UPR activation, and the potential effect of epigenetic inhibitors acting against their HMTs and HDMTs on equilibrating the consequences of excessive ER stress activation.

## 2. Materials and Methods

### 2.1. Cell Cultures and Treatments

Human renal proximal tubular epithelial cells (HK-2 cell line; ATCC CRL-2190; American Type Culture Collection, Manassas, VA, USA) were grown in RPMI 1640 medium (Gibco, Carlsbad, CA, USA) supplemented with 10% FBS, 100 units/mL penicillin, 100 μg/mL streptomycin, 1% ITS (insulin transferrin selenite; 5 μg/mL; Gibco) and hydrocortisone (36 μg/mL; Sigma-Aldrich, St. Louis, MO, USA). To perform the experiments, 6-well culture plates were seeded with 3.5 × 10^5^ cells in complete medium and treated with thapsigargin (Tg, 4 μM; T9033, Sigma) to induce ER stress and UPR activation. The HMTs and HDMs inhibitors used were: GSK16 (EZH2 inhibitor, 2–8 μM, 24 h, S7061); BIX-01294 (G9a inhibitor, 5–15 μM, 24 h, S8006); and GSKJ4 (JMJD3 inhibitor, 5–15 μM, 24 h, S7070) purchased from Selleck Chemicals, Houston, TX, USA, and SD-70 (KDM4C inhibitor, 10–20 μM, 12 h) from Sigma).

### 2.2. Cell Transfection and Gene Silencing

All transfections were carried out using DharmaFECT1 Transfection Reagent (GE Healthcare Dharmacon, Lafayette, CO, USA), following the manufacturer’s instructions. For this purpose, subconfluent HK-2 cells (3 × 10^5^) were plated in 6-well plates and transfected with specific siRNA specific to EZH2 (SI00063959, Qiagen, Hilden, Germany), G9a (SI00091203, Qiagen), JMJD3 (SC93819, Santa Cruz Biotechnology, Dallas, TX, USA), KDM4C (108664, Life Tcehnologies, Carlsbad, CA, USA), and scrambled siRNA (D-001810-10-05, Dharmacon) as a negative control. All siRNAs were used at a range of concentrations between 20 and 80 nM. After transfection, cells were incubated in serum-free medium for a further 48 h, and Tg (4 μM) was added in the final 24 h to induce ER stress activation. Knockdown of each gene was validated by quantitative RT-PCR.

### 2.3. Gene Expression Studies

Total cellular RNA was extracted with a GeneMATRIX Universal RNA purification kit (EURX, Poland), according to the manufacturer’s instructions, and cDNA was synthetized from 1 μg total RNA with the High-Capacity cDNA Reverse Transcription kit (Applied Biosystems, Foster City, CA, USA). Gene expression was measured by quantitative RT-PCR in a MyiQTM Single Color Real-Time PCR Detection System (Bio-Rad, Hercules, CA, USA) using TB Green Premix Ex TaqII (Takara Bio Inc., Kusatsu, Japan). Data were normalized relative to GAPDH RNA expression, and all samples were run in triplicate. Relative mRNA abundance was determined by comparing threshold values and calculated by the 2^−ΔCT^ method (ΔCt: Ct gene test—Ct endogenous control). Specific primers are indicated in Table S1.

### 2.4. Western Blot Analysis

HK-2 cells were isolated in RIPA buffer (Cell Signaling Technologies, Danvers, MA, USA) supplemented with a protease and phosphatase inhibitor cocktail (A32959, Thermo Scientific, Waltham, MA, USA) for 30 min on ice. Proteins were quantified using Bradford protein assay (Bio-Rad) and 30–50 μg of proteins per lane were separated in 10% polyacrylamide-SDS gels, blotted onto 0.45-µm Immobilon-E PVDF membranes (Merck-Millipore, Burlington, MA, USA), and detected by Western blot analysis. Membranes were incubated with specific primary antibodies against ATF4 (11815S, 1:1000; Cell Signaling Technology), XBP1 (NBP2-20917, 1:1000; Novus Biologicals, Littleton, CO, USA), and β-actin (4967S, 1:5000; Cell Signaling Technology) as an endogenous control. Immunoreactive bands were developed using Immobilon Forte Western HRP Substrate (Merck Millipore, MA, USA) and quantified wit Image J software, version 1.53k (NIH, EEUU).

### 2.5. Chromatin Immunoprecipitation Assay

HK-2 cells (0.5–1 × 10^7^ cells per sample) were treated with Tg in the presence or absence of epigenetic inhibitors and fixed with 1% formaldehyde (Sigma) in PBS for 30 min at 4 °C. After that, the reaction was stopped with glycine solution (125 mM, 10 min at 4 °C) and cells were lysed in SDS-lysis buffer (1% de SDS, 10 mM EDTA and 50 mM Tris-HCL, pH 8.1). Chromatin was shared by sonication on a BioRuptor (Diagenode, Liège, Belgium) into 500–1000 bp-long DNA fragments. Next, shared chromatin (100 μg) was diluted into ChIP dilution buffer (0.01% SDS; 1.1% Triton X100; 1.2 mM EDTA; 16.7 mM Tris-HCL, pH 8.1 and 167 mM NaCl) containing protease and phosphatase inhibitor cocktail (Thermo Fisher) and immunoprecipitated with specific antibodies overnight at 4 °C. The antibodies used were specific to the following proteins: EZH2 (49-1043; Thermo Fisher), G9a (ab40542; Abcam, Cambridge, UK), trimethylated H3K27 (H3K27me3, PA5-31817; Thermo Fisher), trimethylated H3K9 (H3K9me3, 05-1242; Merck), acetylated histone H3 (AcH3, 06-599; Merck), acetylated histone 4 (AcH4, 06-598; Merck), and as a negative control, normal rabbit IgG (A300-109A; Bethyl Laboratories, Montgomery, AL, USA). Salmon sperm DNA/Protein A-Agarose beads (Merck) were used to recovered antibody-chromatin complexes (1 h at 4 °C), that were further washed and eluted with elution buffer (1% SDS; 0.1 M NaHCO_2_). After reverse crosslinking and proteinase K treatment, DNA was extracted and analyzed by quantitative RT-PCR using specific primers (Table S1). Chromatin obtained before immunoprecipitation was used as an input control. Data are expressed as the fold enrichment (FE) of each specific antibody relative to the negative control (normal IgG); FE = 2 exp − [ΔCt (specific antibody) − ΔCt (normal IgG)], being ΔCt = Ct (bound) − [Ct (input) − log2 (Input dilution factor)]. To analyze the interaction between EZH2 and G9a and their binding to the chromatin, a ChIP assay was carried out using a specific antibody against EZH2, and after washing the antibody-chromatin complexes, proteins were eluted and analyzed by Western blot using anti-EZH2 or anti-G9a antibodies.

### 2.6. Statistical Analysis

Data are summarized as the mean ± standard error of the mean (SEM) of at least three independent assays. Normal distribution of the data was tested using the Shapiro–Wilk test. Groups were compared using Student’s paired-samples t-test (for normally distributed data) or the Mann–Whitney U test (for non-normally distributed data). Statistical analyses were performed using the IBM SPSS Statistics v.23.0 application (IBM Corp, Armonk, NY, USA) and GraphPad Prism 8.0 (GraphPad Software, San Diego, CA, USA). In all cases, a value of *p* < 0.05 was considered to be statistically significant.

## 3. Results

### 3.1. Blockage of the G9a and EZH2 HMTs Induces Expression of ATF4 and XBP1

**Figure 2:**
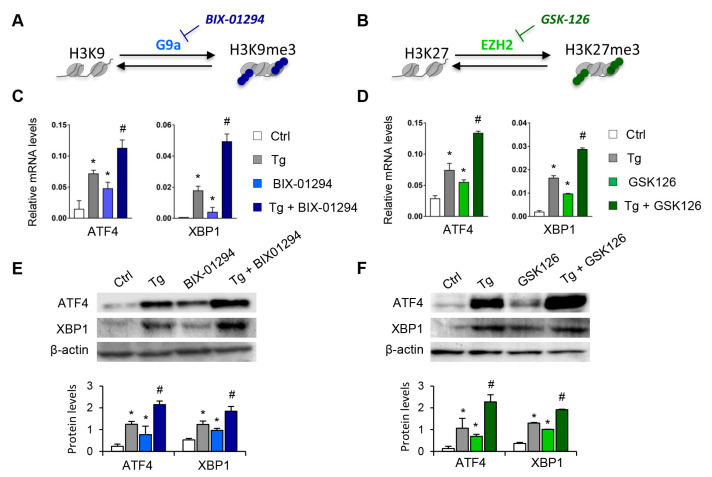
Pharmacological inhibition with BIX-01294 and GSK126 induces the expression of ATF4 and XBP1 in tubule epithelial cells. (**A**,**B**) Scheme of the effect of the BIX-01294 and GSK126 inhibitors on blocking the G9a and EZH2 histone methyltransferase (HMT) enzymes and on inducing modifications of the methylation of K9 (Lys 9) and K27 (Lys 27) of histone 3 (H3), respectively. Human renal tubular epithelial (HK-2) cells were treated with BIX-01294 (G9a inhibitor, 15 μM, 24 h) or GSK126 (EZH2 inhibitor, 8 μM, 24 h) in the absence or presence of thapsigargin (Tg, 4 μM, 24 h). Cells treated with DMSO were used as a negative control. Gene expression levels of ATF4 and XBP1 were analyzed by quantitative RT-PCR (**C**,**D**); protein levels were assessed by Western blotting (**E**,**F**). GAPDH and β-actin were used as controls. Data are presented as the mean ± SEM of at least three independent experiments. * *p* < 0.05 vs. control (DMSO-treated cells); # *p* < 0.05 vs. Tg-induced cells.

To determine whether the G9a and EZH2 epigenetic enzymes are involved in the regulation and activation of the UPR pathway, HK-2 cells were treated with pharmacological inhibitors specific to both molecules, GSK126 (EZH2 inhibitor) or BIX-01294 (G9a inhibitor), in the presence or absence of thapsigargin (Tg, 4 μM). Tg is an inhibitor of SERCA (sarco/endoplasmic reticulum Ca_2_^+^ ATPase) activity, which induces the activation of the three major UPR branches, triggering the expression of ATF4, XBP1, and ATF6. Treatment with both inhibitors, in an independent mode, increases the transcriptional levels of ATF4 and XBP1 genes in a dose-dependent manner (Figure 2A–D and Figure S1). Moreover, this effect on ATF4 and XBP1 expression was in addition, in both cases, to that observed after ER stress induction with Tg (Figure 2C,D). No changes in the ATF6 transcription factor were detected (data not shown). Likewise, G9A and EZH2 inhibitors enhanced the ATF4 and XBP1 protein levels, even higher than induction with Tg (Figure 2E,F).

**Figure 3:**
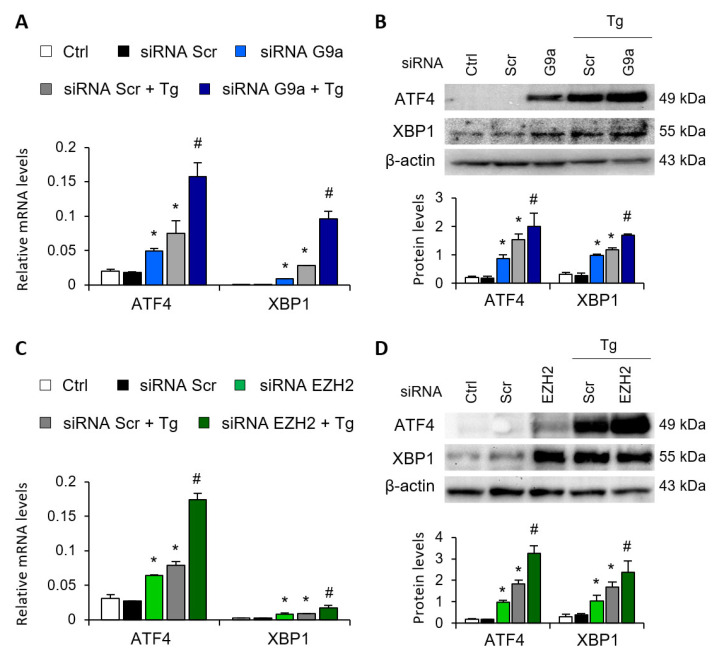
G9a and EZH2 disruption favors ATF4 and XBP1 upregulation in renal tubular epithelial cells. HK-2 cells were transfected with specific G9a siRNA (**A**,**B**), EZH2 siRNA (**C**,**D**) or nonspecific scramble (Scr) siRNA (80 nM) for 48 h. Tg (4 μM) was added in the final 24 h to induce UPR activation. Gene expression (**A**,**C**) and protein (**B**,**D**) levels of ATF4 and XBP1 were assayed by quantitative RT-PCR and Western blotting, respectively. GAPDH and β-actin were used as controls. Results are expressed as the mean ± SEM of three independent experiments. * *p* < 0.05 vs. siRNA Scr; # *p* < 0.05 vs. siRNA Scr + Tg (cells transfected with siRNA Scr and treated with Tg).

Gene-silencing assays were performed to corroborate the role of G9a and EZH2 in the transcriptional regulation of ATF4 and XBP1. To this end, HK-2 cells were transfected with small interfering RNA (siRNA) that specifically targeted G9a and EZH2. In a similar way to that previously described with the specific treatments, specific knockout of the two HMTs, G9a and EZH2, increased the ATF4 and XBP1 transcriptional expression in the absence of Tg (Figure 3A,C) and in a dose-dependent manner (Figure S2), whilst no effect was detected with the control siRNA. In a similar way to that previously observed with the epigenetic inhibitors, the combined treatment with Tg yielded a greater increase in the transcriptional and protein levels of ATF4 and XBP1 than when the two HMTs were independently inhibited (Figure 3A–D). Thus, pharmacological or specific inhibition of both G9a and EZH2 HMTs enables ATF4 and XBP1 transcription even in the absence of UPR pathway activators.

### 3.2. G9a and EZH2 HMTs Bind to ATF4 and XBP1 Promoter, Allowing the Formation of a Repressive Chromatin Structure

**Figure 4:**
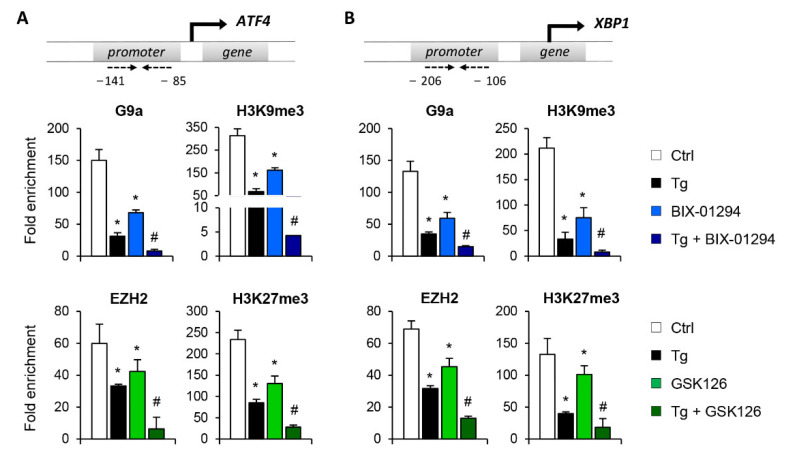
Treatment with BIX-01294 and GSK126 inhibits the direct binding of G9a and EZH2 to the ATF4 and XBP1 promoters, reducing the presence of repressive histone marks. HK-2 cells were treated with BIX-01294 (G9a inhibitor, 15 μM, 24 h) or GSK126 (EZH2 inhibitor, 8 μM, 24 h) in the absence or presence of thapsigargin (Tg, 4 μM, 24 h). Cells treated with DMSO or Tg were used as negative and positive controls, respectively. ChIP assays were performed using specific antibodies against G9a, H3K9me3, EZH2 and H3K27me3, and normal rabbit IgG was used as a negative control. Enrichment of the DNA sequence associated with these HMTs and repressive marks in the promoter region of ATF4 (**A**) and XBP1 (**B**) genes were determined by quantitative RT-PCR using specific primers (dashed arrows). Data are shown as the mean ± SEM of three independent experiments and each RT-PCR was run in triplicate. Results are represented as the fold enrichment of each specific antibody relative to negative control (normal rabbit IgG). * *p* < 0.05 vs. control and # *p* < 0.05 vs. cells treated with BIX-01294 or GSK126.

Having demonstrated that G9a and EZH2 are involved in repressing the ATF4 and XBP1 transcription factors, the following step was to determine whether it is mediated by the direct binding of these HMTs to the regulatory regions of both genes. To achieve this, chromatin immunoprecipitation (ChIP) assays using specific antibodies against G9a and EZH2 were performed in the ATF4 and XBP1 promoter region of untreated HK-2 cells or treated with BIX-01294 (G9a inhibitor) or GSK126 (EZH2 inhibitor) in the presence or absence of Tg. Results revealed increased enrichment of G9a and EZH2 in the ATF4 and XBP1 promoter in control cells (untreated) (Figure 4A,B). However, the binding of G9a and EZH2 was significantly reduced after treatment with BIX-01294 or GSK126, respectively (Figure 4A,B). The recruitment of both HMTs was even lower when it was combined with Tg. Similar results were observed when the enrichment of the H3K9me3 and H3K27me3 repressive marks, catalyzed by G9a and EZH2 respectively, was analyzed in the same promoter region. A high level of enrichment of both histone marks was detected in untreated HK-2 cells, which was related to the low level of expression of these genes reported above (Figure 4A,B). After treatment with the BIX-01294 or GSK16 inhibitor, in the presence or absence of Tg, the enrichment of both repressive marks was significantly reduced, in association with the previously described induced expression of ATF4 and XBP1 (Figure 4A,B). These findings confirm that the G9a and EZH2 enzymes are recruited to the promoter region of the ATF4 and XBP1 genes, increasing the presence of the H3K9me3 and H3K27me3 repressive histone modifications, and favoring their transcriptional repression with it.

It has been previously reported that epigenetic complexes that mediate histone methylation such as PRC2 (Polycomb repressive complex 2), whose catalytic subunit is EZH2, can recruit histone deacetylases (HDACs) to maintain a repressive state [21]. To explore the acetylation dynamics in the ATF4 and XBP1 genes, the presence of acetylated histone H3 (AcH3) or H4 (AcH4) was determined by ChIP assay in the same regulatory regions. Data showed an enrichment in the AcH3 and AcH4 levels after pharmacological inhibition of G9a and EZH2, in the presence or absence of Tg (Figure S3). However, in untreated cells the acetylation levels in histones were almost undetectable. Overall, the binding of G9a and EZH2 HMTs to the ATF4 and XBP1 genes is essential to maintain a repressive chromatin structure that prevents the exacerbated expression of these transcription factors under physiological conditions.

### 3.3. Crosstalk between G9a and EZH2 Represses Expression of ATF4 and XBP1

**Figure 5:**
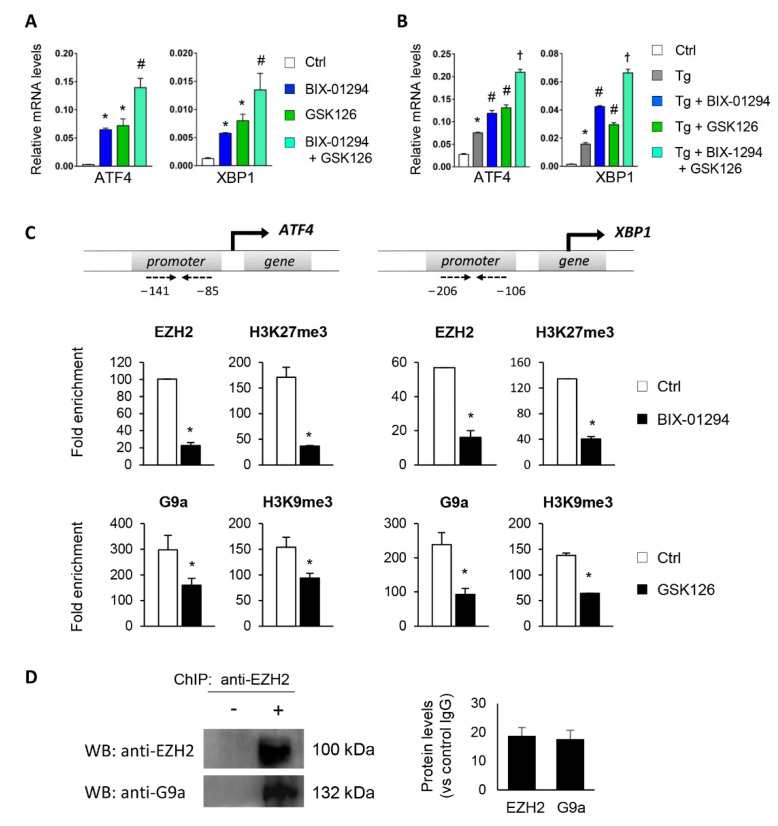
Interaction between G9a and EZH2 regulates ATF4 and XBP1 transcriptional repression. HK-2 cells were treated with BIX-01294 (G9a inhibitor, 15 μM, 24 h), GSK126 (EZH2 inhibitor, 8 μM, 24 h) or the combination of the two, in the absence (**A**) or presence (**B**) of thapsigargin (Tg, 4 μM, 24 h). Cells treated with DMSO were used as a negative control. Gene expression levels of ATF4 and XBP1 were analyzed by quantitative RT-PCR using GAPDH as an internal control. * *p* < 0.05 vs. control (DMSO-treated cells); # *p* < 0.05 vs. Tg-induced cells; and † *p* < 0.05 vs. treatment with BIX-01294 or GSK126. (**C**) ChIP assay was carried out in HK-2 cells treated with BIX-01294 (15 μM, 24 h) or GSK126 (8 μM, 24 h) using specific antibodies for EZH2 or H3K9me3 and G9a and H3K27me3, respectively. Enrichment in the promoter region of ATF4 and XBP1 genes was determined by quantitative RT-PCR using specific primers (dashed arrows). * *p* < 0.05 vs. control. (**D**) ChIP assay in untreated HK-2 cells with anti-EZH2 antibody and further Western blotting with EZH2 and G9a antibodies. Data are shown as the mean ± SEM of two independent experiments.

To evaluate whether G9a and EZH2 can act together to regulate ATF4 and XBP1, a combined treatment with BIX-01294 and GSK126 was assessed under basal conditions or following UPR induction with Tg. Results showed that the combination of both inhibitors had a greater increase in ATF4 and XBP1 transcriptional levels than each individual treatment (Figure 5A), suggesting that G9a and EZH2 act collaboratively to regulate these genes. The same pattern was observed in Tg-stimulated HK-2 cells (Figure 5B).

Furthermore, to explore the crosstalk between the two HMTs, ChIP assays were performed on the ATF4 and XBP1 promoter using antibodies against EZH2 and H3K27me3 in HK-2 cells treated with BIX-01294 (G9a inhibitor) and vice versa. Treatment with BIX-01294 significantly reduced the recruitment of EZH2 and the H3K27me3 levels in the regulatory region of ATF4 and XBP1 (Figure 5C). Similarly, treatment with the EZH2 inhibitor, GSK126, reduced the presence of G9a and H3K9me3 (Figure 5C), confirming that the two enzymes work in collaboration to ensure the epigenetic silencing of ATF4 and XBP1.

We also checked whether EZH2 and G9a could collaborate in binding to chromatin and to produce further gene regulation by carrying out a ChIP assay using an anti-EZH2 antibody in untreated HK-2 cells. The immunoprecipitated protein–DNA complex was then used to detect G9a and EZH2 proteins by Western blot. Results showed that G9a coimmunoprecipitated with EZH2 in untreated HK-2 cells (Figure 5D), suggesting that the two HMTs physically interact with each other to induce trimethylation of the H3K9 and H3K27 histone residues.

### 3.4. Blockage of the KDM4C and JMJD3 Histone Demethylases Impairs the ATF4 and XBP1 Expression under UPR Activation

**Figure 6:**
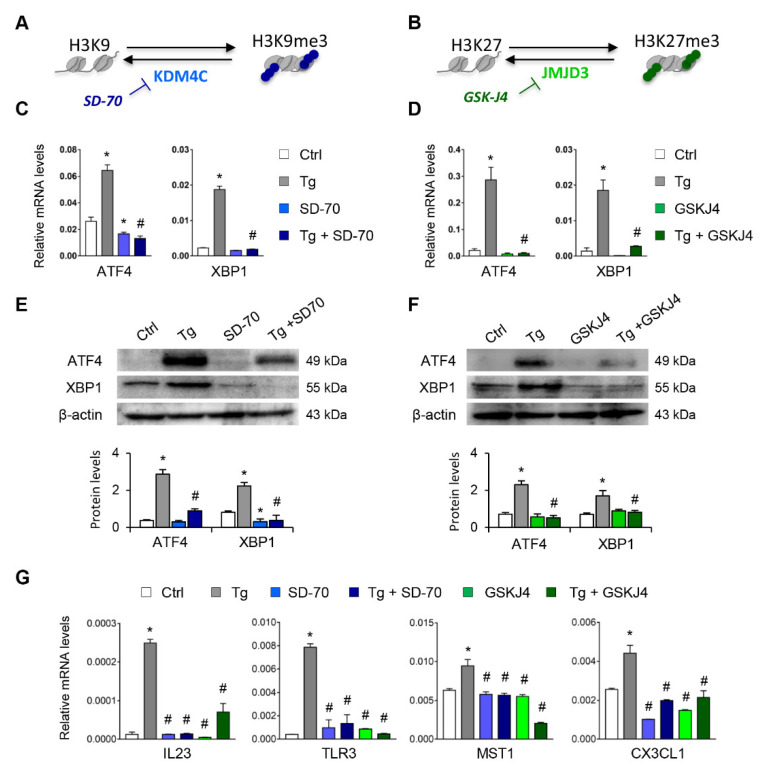
Treatment with SD-70 and GSKJ4 reverses the ATF4 and XBP1 expression induced by UPR activation. (**A**,**B**) Scheme of the effect of the SD-70 and GSKJ4 inhibitors on blocking the KDM4C and JMJD3 histone demethylase (HDM) enzymes and inducing H3K9 and H3K27 trimethylation, respectively. Human renal tubular epithelial cells (HK-2 cells) were treated with SD-70 (KDM4C inhibitor, 20 μM, 12 h) or GSKJ4 (JMJD3 inhibitor, 15 μM, 24 h) in the absence or presence of thapsigargin (Tg, 4 μM, 24 h). Cells treated with DMSO were used as a negative control. Gene expression levels of ATF4 and XBP1 were analyzed by quantitative RT-PCR (**C**,**D**) and protein levels by Western blotting (**E**,**F**). (**G**) Gene expression of XBP1-dependent (IL23, TLR3), ATF4-dependent (MST-1), and XBP1/ATF4-dependent (CX3CL1) genes. GAPDH and β-actin were used as controls for RT-PCR and Western blotting, respectively. Data are presented as the mean ± SEM of at least three independent experiments. * *p* < 0.05 vs. control (DMSO-treated cells); # *p* < 0.05 vs. Tg-induced cells.

Having established that the H3K9me3 and H3K27me3 histone mark levels are essential for suppressing expression of ATF4 and XBP1, we analyzed whether the blockage of their histone demethylases, KDM4C and JMJD3, respectively, might be useful for inhibiting the expression of these transcription factors under UPR activation (Figure 6A,B). Treatments with SD-70 (KDM4C inhibitor, 20 μM) or GSKJ4 (JMJD3 inhibitor, 15 μM) significantly reduced the levels of ATF4 and XBP1 expression in HK-2 cells (Figure S4). This inhibition was also observed in HK-2 cells stimulated with Tg at transcriptional (Figure 6C,D) and protein (Figure 6E,F) levels similar to or below those of control cells. Moreover, the transcriptional levels of genes regulated by ATF4 (MST1), XBP1(IL23 and TLR3), or both (CX3CL1) were also downregulated after treatment with SD-70 and GSKJ4 and under UPR activation (Figure 6G), confirming that the HDM inhibitor reduced the expression not only of the ATF4 and XBP1 transcription factors, but also of their downstream regulated genes.

**Figure 7:**
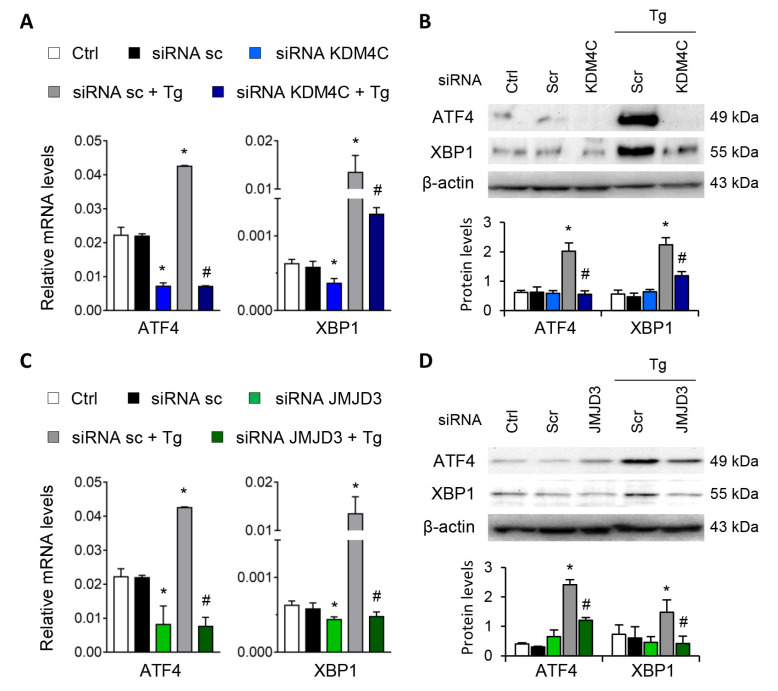
Specific inhibition of KDM4C and JMJD3 limits the ATF4 and XBP1 overexpression induced by UPR activation**.** HK-2 cells were transfected with specific KDM4C siRNA (**A**,**B**), JMJD3 siRNA (**C**,**D**), or nonspecific scramble (Scr) siRNA (80 nM) for 48 h. Tg (4 μ M) was then added in the final 24 h to induce UPR activation. Gene expression (**A**,**C**) and protein levels (**B**,**D**) of ATF4 and XBP1 were assayed by quantitative RT-PCR and Western blotting, respectively. GAPDH and β-actin were used as control. Results are expressed as the mean ± SEM of three independent experiments. * *p* < 0.05 vs. siRNA Scr; # *p* < 0.05 vs. siRNA Scr + Tg (cells transfected with siRNA Scr and treated with Tg).

Similar findings emerged after specific knockdown of KDM4C and JMJD3 with specific siRNA, in absence of Tg in a dose-dependent manner (Figure S5). Moreover, the induction of ATF4 and XBP1 by Tg was almost eliminated after blockage of KDM4C and JMJD3, but not with the scramble siRNA (Figure 7A–D).

**Figure 8:**
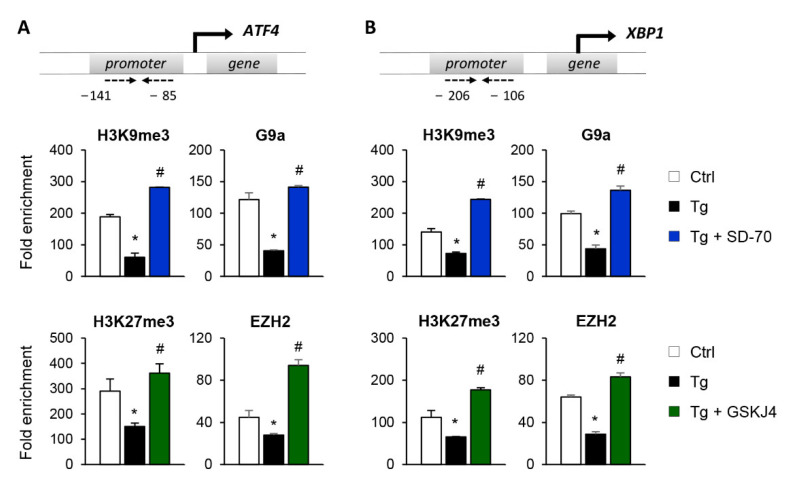
Inhibition of the KDM4C and JMJD3 histone demethylases restores the epigenetic changes induced by UPR activation. HK-2 cells were treated with SD-70 (KDM4C inhibitor, 20 μM, 24 h) or GSKJ4 (JMJD3 inhibitor, 15 μM, 24 h) in the presence of thapsigargin (Tg, 4 μM, 24 h). Treatment with DMSO and Tg were used as negative and positive controls. ChIP assays were performed using specific antibodies against H3K9me3,H3K27me3, G9a, and EZH2. Enrichment in the promoter region of ATF4 (**A**) and XBP1 (**B**) genes were determined by quantitative RT-PCR using specific primers (dashed arrows). Data are presented as the mean ± SEM of two or three independent experiments and each RT-PCR was run in triplicate. Results are represented as the fold enrichment of each specific antibody relative to a negative control (normal rabbit IgG). * *p* < 0.05 vs. control and # *p* < 0.05 vs. cells treated with Tg.

To establish whether the reduced expression of ATF4 and XBP1 after SD-70 and GSKJ4 treatment is due to an enhanced presence of the H3K9me3 and H3K27me3 repressive marks at the regulatory region of these genes, we performed ChIP assays using specific antibodies against these histone modifications. The results confirmed that treatment with Tg reduces the enrichment of H3K9me3 and H3K27me3 at the regulatory region of the ATF4 and XBP1 genes, demonstrating this to be associated with their higher levels of expression (Figure 8A,B). However, pharmacological inhibition of the KDM4C and JMJD3 HDMs with SD-70 and JMJD3, respectively, restored the presence of these repressive marks to similar levels to those of controls even when the UPR was activated with Tg (Figure 8A,B). The increased levels of H3K9me3 and H3K27me3 observed after treatment with SD-70 and GSKJ4, respectively, and under ER stress, are associated with a higher recruitment of the G9a and EZH2 HMTs at the promoter region of ATF4 and XBP1 (Figure 8A,B). Therefore, these findings strongly suggest that pharmacological inhibition of JMJD3 and KDM4C allows maintaining a repressive chromatin structure in ATF4 and XBP1 genes, and consequently prevents activation of the UPR pathway under ER stress conditions.

## 4. Discussion

Epigenetic dynamics mediated by chemical modifications in the histone tails (acetylation and methylation, among others), and DNA methylation help modulate chromatin structure and regulate gene transcription. The epigenetic landscape of each gene, mainly mediated by histone modifications, will induce or repress its gene transcription to respond efficiently to different stimuli and to the environment. Thus, changes in chromatin dynamics make it possible to adapt the transcriptional profile of each cell in response to stress. As a consequence of the loss of ER proteostasis, tubular cells need to induce a rapid expression of genes involved in the UPR activation to solve the problem. In this study, we elucidate the epigenetic changes mediated by histone methylation required for proper UPR activation triggered by ER stress.

**Figure 9:**
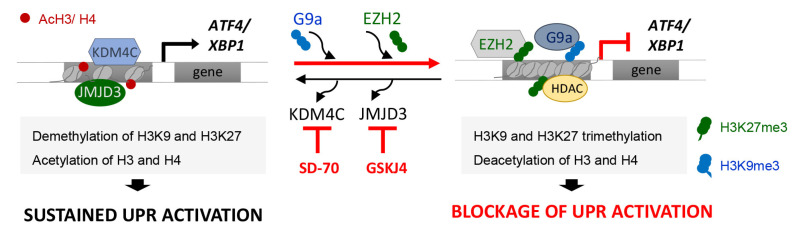
Blockage of the KDM4C and JMJD3 HDMs allows the inhibition of ATF4 and XBP1 expression under UPR activation in renal tubular cells. Modulation of the methylation levels in the H3K9 and H3K27 histone marks, mediated by the G9a and EZH2 histone methyltransferase (HMTs) enzymes or the KDM4C and JMJD3 histone demethylases (HDMs), enables the expression of the ATF4 and XBP1 transcription factors and their downstream signaling to be regulated. Treatment with the SD-70 and GSKJ4 epigenetic inhibitors (red lines) leads to an increased recruitment of HMTs and enhanced H3K9 and H3K27 methylation levels at ATF4 and XBP1 genes, reducing their expression and tip the balance toward reduced signaling mediated by these transcription factors, and thereby the renal damage under sustained ER stress.

Our study reveals that the G9a-mediated H3K9me3 and EZH2-mediated H3K27me3 modifications are key to maintaining the transcriptional repression of ATF4 and XBP1 genes in renal tubular epithelial cells under sustained ER stress (Figure 9). Accordingly, we present evidence that treatment with the KDM4C and JMJD3 histone demethylases inhibitors, SD-70 and GSKJ4 respectively, represses the ATF4 and XBP1 expression induced by UPR activation, which causes the downmodulation of genes regulated by these transcription factors (Figure 9). Thus, these results might be useful for establishing a new therapeutic strategy and blocking the ER stress response triggered during renal damage.

The addition of methyl groups to the H3K9 and H3K27 histone residues is mediated by the G9a and EZH2 HMTs. G9a and Suv39h1 catalyze the mono-, di-, and trimethylation reactions on H3K9 [22] and it is known to be involved in H3K27 methylation in vivo, suggesting a more heterologous role [23]. Additionally, EZH2 is responsible for the methylation of histone H3 at lysine 27 (H3K27), which leads to chromatin compaction [24]. Both HMTs are known to have abnormal expression and activity in several pathological processes associated with renal damage [25,26,27], and even in transplanted and aging kidneys [28]. The activation of several intracellular signaling pathways that leads to renal fibrosis and the epithelial-mesenchymal transition is modulated by EZH2 and H3K27me3. In fact, EZH2 gene silencing or pharmacological inhibition is associated with the reduced expression of EGFR and PDGFR receptors, downmodulation of the TGF-β/Smad3, AKT, and ERK1/2 pathways, and the Snail and Twist factors [29]. Irufuku et al. [27] showed that G9a inhibition with BIX-01294 reduces the expression of TGF-β-induced pro-fibrotic markers (α-SMA and fibronectin) and, more interestingly, restores the expression of klotho.

Epigenetic dynamics mediated by histone methylation in the kidney has been little studied in the context of cellular stress and distinct roles of EZH2 have been reported depending on the regulated gene or cell type. So, EZH2 inhibition attenuated the increase of ROS in renal tubular cells and during the I/R injury [30,31]. Likewise, EZH2 inhibition protects against hyperoxaluria-induced kidney injury by blocking the production of ROS and CaOx crystal deposition reduction [32]. Nevertheless, EZH2 blockage induces podocyte damage mediated by TxnIP expression and enhances oxidative stress [33,34]. These differences could also be due to the different inhibitors used in each study. Most of the studies reported here analyzed the effect of 3-DZNep, a potent inhibitor of SAH hydrolase that reduces SAM levels in cells, ultimately causing global inhibition of histone methylation, including the reactions catalyzed by EZH2 [35]. However, other inhibitors, such as GSK126, which we used in this work, are specific inhibitors targeting the catalytic site of EZH2, exclusively.

The involvement of G9a and EZH2 and their methylation dynamics in regulating the UPR pathway has only been explored in a tumor context, showing that inhibition of both HMTs promotes apoptosis and autophagy by preventing tumor progression. Demethylation of H3K9 and H3K27 in IRE1α and PERK genes induces ER stress/UPR activation, compromising the tumor growth in gastric, diffuse B-cell lymphoma, and colorectal cancer [36,37,38]. To the best of our knowledge, this study is the first to analyze the epigenetic dynamics involved in renal UPR activation. We determined that G9a and EZH2 bind to the ATF4 and XBP1 promoter and cooperate each other and with HDACs to repress the expression of these transcription factors induced by the UPR activator, thapsigargin. Previous studies reported that EZH2 works cooperatively with HDACs to form long protein complexes aimed at facilitating a closed chromatin structure, and impair gene transcription [39]. The physical interaction between G9a and EZH2 and its combined effect was shown in the silencing of several cytokines, chemokines, and development genes [40,41,42,43,44]. G9a can methylate H3K27 in vitro and in vivo, suggesting that there is crosstalk between the two enzymes and a degree of plasticity in the methylation patterns [45]. According to this, our results showed that interdependent crosstalk between EZH2 and G9a is required to bring about the epigenetic silencing of ATF4 and XBP1 in renal tubular cells.

Sustained activation of the ER stress and development of a maladaptive UPR have been associated with aggravated renal damage in response to diverse stimuli or damage signals. This leads to a continuous activation of the PERK- and IRE1α-mediated signaling pathways, which culminates in the increased overexpression of genes regulated by ATF4 and XBP1, respectively. In the kidney, these alterations are associated with greater apoptosis, oxidative stress, proteinuria, inflammation, and fibrosis, which impair the restoration of renal function. Thus, inhibition of the UPR signaling pathways or the triggered downstream processes have been proposed to reduce the disease progression [46,47]. It is important to note that ATF6-mediated signaling also helps restore ER homeostasis but its contribution to renal pathology is less clear. In our study, the modulation of H3K9 and H3K27 methylation profiles does not induce changes in ATF6 expression. However, ATF6 can specifically bind to the XBP1 promoter, enhancing its expression and splicing by IRE1α in response to ER stress [48]. Therefore, XBP1 can function in a more sustained way, with extremely high levels of ER stress making its blockage more effective, thereby preventing the pathological processes from being triggered.

In this study, we provide evidence that by arresting the histone demethylases responsible for removing the methyl groups from H3K9 and H3K27 residues, ATF4 and XBP1 gene silencing can be maintained even under ER stress/UPR activation. These findings are consistent with the previous observation that the expression of KDM4C reduces H3K9me3 levels in the ATF4 promoter, favoring its expression and transactivation to reprogram amino acid metabolism and cell proliferation in neuroblastoma and osteosarcoma cells [49]. Until now, few studies had analyzed the effect of JMJD3 inhibition in renal pathology, but some had begun to show its effect to reduce the expression of inflammatory genes (IL6, IL8, IL1β) and prevent p53-dependent apoptosis ameliorating renal dysfunction in diabetic kidney disease [50]. In mesangial cells, TGF-β upregulates JMJD3, reducing the H3K27me3 levels of Ctgf, Serpine 1, and Ccl2 and, reciprocally, boosting its expression [51]. The JMJD3 inhibitor, GSKJ4, prevents podocyte dedifferentiation and attenuating glomerular damage through reduced expression of the Notch ligand Jagged1 (JAG1) [20]. Nevertheless, it has recently been described that JMJD3 is required to limit the activation of diverse profibrotic signaling pathways in UUO mice and fibroblast cells [52]. Despite the known function of G9a HTM in renal pathology, the outcome of KDM4C blockage or inhibition has yet to be established.

Of note, H3K27me3 and H3K9me3 histone marks regulate a broad spectrum of target genes in the renal cells. For instance, it is known that proliferation and differentiation of epithelial tubular cells are critical steps of renal regeneration during AKI and that histone methylation might also be involve in these processes [53]. Moreover, the epigenetic landscape is cell-type-specific and various cell types can be damaged in the whole kidney depending on the kidney disease [54]. Thus, further studies in mouse models are needed to evaluate the overall consequences of epigenetic reprogramming in the kidney, and to fully explore whether their inhibition in renal cells may entail the generation of side effects. Nonetheless, it is highlighted that epigenetic modifications are dynamic, change with time, and can be reversible acting in the involved enzymes, suggesting that the potential pharmacological alteration of these epigenetic landscapes could be exploited in an interested fashion by their use in a delimited time and space.

## 5. Conclusions

Taken together, our results indicate that epigenetic dynamics mediated by the H3K9 and H3K27 histone methylation are key to modulating the expression of the ATF4 and XBP1 transcription factors and shed new light on the modulation of the exacerbated ER stress response and the underlying pathological consequences. Pharmacological inhibition of the HDMs involved (KDM4C and JMJD3) could be useful for abrogating the pathological consequences triggered by maladaptive UPR activation during renal damage, though in vivo studies in mouse models of kidney damage are required to further validate these results and the overall consequences in the kidney.

## Data Availability

Data are contained within the article or Supplementary Materials.

## Supplementary Materials

The following supporting information can be downloaded at: https://www.mdpi.com/article/10.3390/antiox11071355/s1, Figure S1: Treatment with BIX-01294 and GSK126 induces ATF4 and XBP1 expression in a dose-dependent manner; Figure S2: Specific blockage of G9a and EZH2 leads to the upregulation of ATF4 and XBP1 in a dose-dependent manner; Figure S3: Treatment with BIX-01294 and GSK126 increases H3 and H4 acetylation levels in the promoter region of ATF4 and XBP1, enhancing their expression; Figure S4: Treatment with SD-70 and GSK126 reduces ATF4 and XBP1 expression in a dose-dependent manner. HK-2 cells were treated with SD-70 (KDM4C inhibitor) or GSKJ4 (EZH2 inhibitor) at different doses for 12 or 24 h, respectively; Figure S5: Specific blockage of KDM4C and JMJD3 leads to the upregulation of ATF4 and XBP1 in a dose-dependent manner; Table S1: Primers for quantitative RT-PCR and ChIP assay.
