# Supplementary material for: Demethylation of H3K9 and H3K27 Contributes to the Tubular Renal Damage Triggered by Endoplasmic Reticulum Stress
Source: Antioxidants (Basel). 2022 Jul 12;11(7):1355. doi: 10.3390/antiox11071355 (PMC9312208; doi:10.3390/antiox11071355)
Supplement: Supplementary file 1 [file antioxidants-11-01355-s001.zip › antioxidants-1797555-supplementary.pdf]

Supplementary materials

Table S1. Primers for quantitative RT-PCR and ChIP assay.

Gene	Gene accession number	Forward primers 5´-3	Reverse primers 5´-3
		RT-PCR	
ATF4	NM_001675.4	CTTGATGTCCCCCTTCGACC	CTTGTCGCTGGAGAACCCAT
CX3CL1	NM_002996.6	CACCACGGTGTGACGAAATG	TCTCCAAGATGATTGCGCGT
EZH2	NM_004456.5	GCGACTGAGACAGCTCAAGA	TGCACAGGCTGTATCCTTCG
EHMT2 (G9a)	NM_025256.7	CCCAACTCTCTACTGGGCTG	TCCCCCAAAGAGCCATGAAC
GAPDH	NM_002046.7	TGCCATGGGTGGAATCATATTGGA	TCGGAGTCAACGGATTTGGTCGT
IL23A	NM_016584.3	GACCCACAAGGACTCAAGGAC	ATGGGGCTATCAGGGAGTAGAG
KDM6B (JMJD3)	NM_001080424.2	TCCGTTTGTGCTCAAGGTGT	GAGCACGATAGTGAGGAGGC
KDM4C	NM_001146696.2	TTTTCCCAAGCAGCTCCCAA	TTCTCCAGCCTCCTGGGTTA
MST1	NM_020998.4	ATACCATGGCCAAGCGGAAT	TCAGCATAAGGGGGCTTTCC
TLR3	NM_003265.3	AGATTACCAGCCGCCAACTT	GCTCATTGTGCTGGAGGTTC
XBP1s	NM_001079539.2	GCTGAGTCCGCAGCAGGT	CTGGGTCCAAGTTGTCCAGAAT
		ChIP assay	
ATF4	NC_000022.11	GTTGGCATGAAGCCCTCTTGAATAA	AGAGTGCTGTAGCTGTGTGTTC
XBP1	NG_012266	ACATGCTAGCCAAGGCTCTAGT	GCAAAACTAAATGTAGCAGGGTAGT

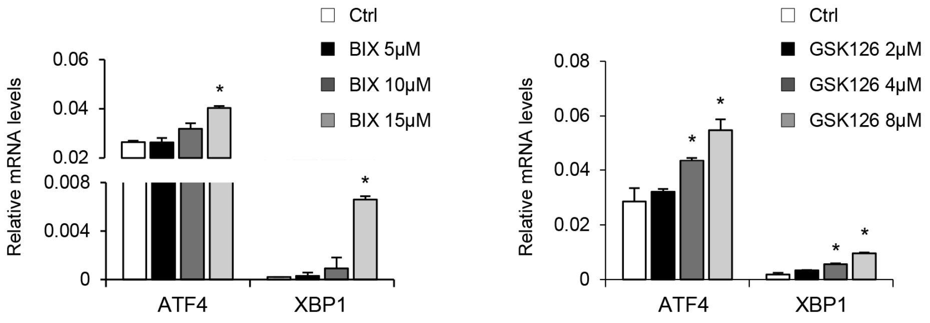

Figure S1. Treatment with BIX-01294 and GSK126 induces ATF4 and XBP1 expression in a dose-dependent manner. HK-2 cells were treated with BIX-01294 (G9a inhibitor) or GSK126 (EZH2 inhibitor) at different doses for 24 h. Cells treated with DMSO were used as a negative control. Gene expression levels of ATF4 and XBP1 were analyzed by quantitative RT-PCR using GAPDH as internal control. Data are represented as mean ± SEM of three independent experiments. *p < 0.05 vs control (DMSO-treated cells).

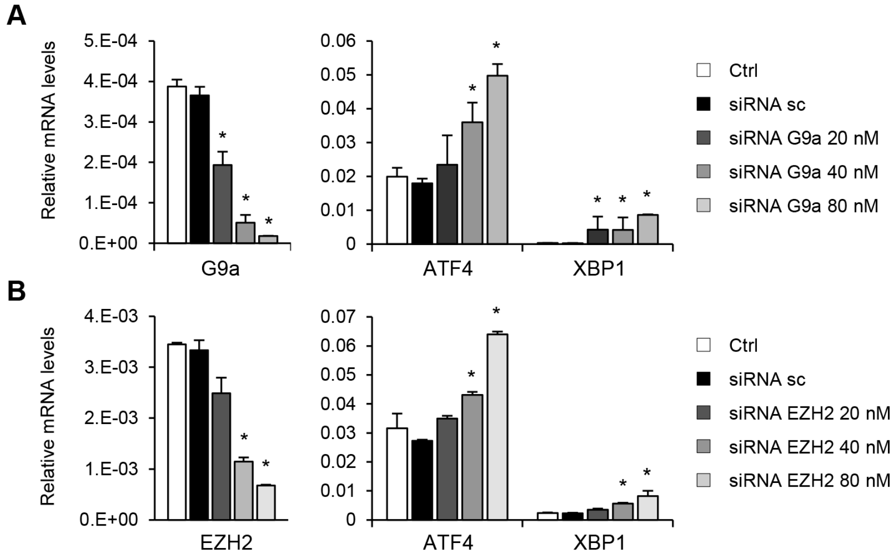

Figure S2. Specific blockage of G9a and EZH2 leads to the upregulation of ATF4 and XBP1 in a dose-dependent manner**.** HK-2 cells were transfected with specific G9A siRNA (A) and EZH2 siRNA (B) or nonspecific scramble (Scr) siRNA at different doses for 48 h. Gene expression was assayed by quantitative RT-PCR using GAPDH as an internal control. Results are expressed as the mean ± SEM of three independent experiments. *p < 0.05 vs siRNA Scr.

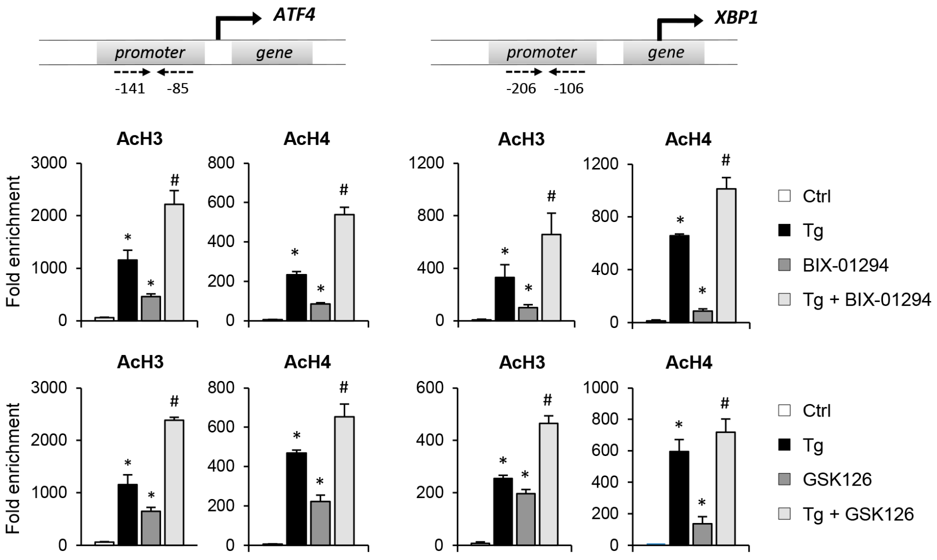

Figure S3. Treatment with BIX-01294 and GSK126 increases H3 and H4 acetylation levels in the promoter region of ATF4 and XBP1, enhancing their expression**.** HK-2 cells were treated with BIX-01294 (G9a inhibitor, 15 μM, 24 h) or GSK126 (EZH2 inhibitor, 8 μM, 24 h) in the absence or presence of thapsigargin (Tg, 4 μM, 24 h). Cells treated with DMSO or Tg were used as negative and positive controls, respectively. ChIP assays were performed using specific antibodies against acetylated histone 3 (AcH3) and histone 4 (AcH4), and normal rabbit IgG was used as a negative control. Enrichment in the promoter region of ATF4 and XBP1 genes were determined by quantitative RT-PCR using specific primers (dashed arrows). Data are shown as the mean ± SEM from three independent experiments; each RT-PCR was run in triplicate.

Results are presented as the fold enrichment of each specific antibody relative to the negative control (normal rabbit IgG). *p < 0.05 vs control and # p < 0.05 vs cells treated with BIX-01294 or GSK126.

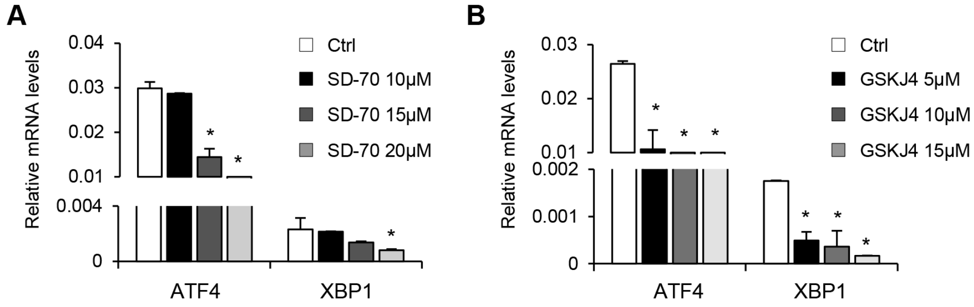

Figure S4. Treatment with SD-70 and GSK126 reduces ATF4 and XBP1 expression in a dose-dependent manner. HK-2 cells were treated with SD-70 (KDM4C inhibitor) or GSKJ4 (EZH2 inhibitor) at different doses for 12 or 24 h, respectively. Cells treated with DMSO were used as a negative control. Gene expression levels of ATF4 and XBP1 were analyzed by quantitative RT-PCR using GAPDH as an internal control. Data are presented as the mean ± SEM of three independent experiments. *p < 0.05 vs control (DMSO-treated cells).

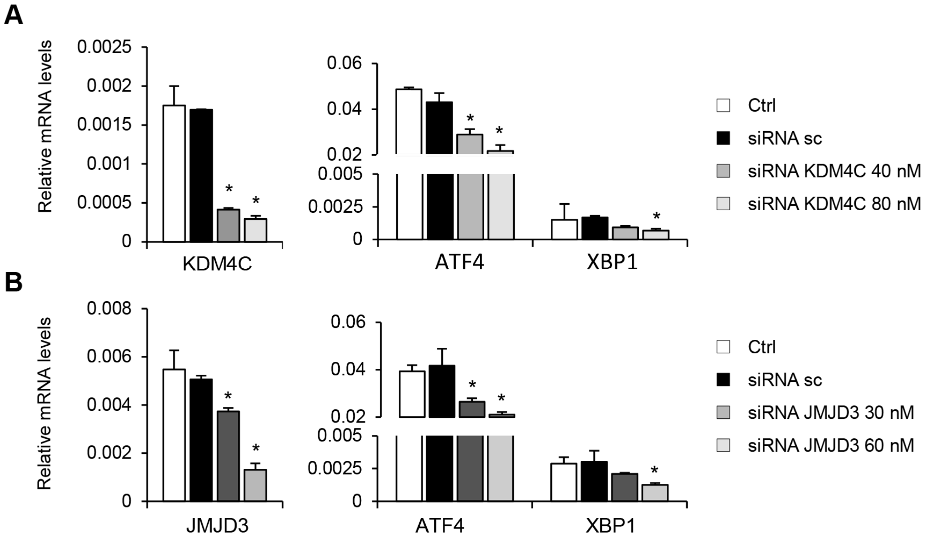

Figure S5. Specific blockage of KDM4C and JMJD3 leads to the upregulation of ATF4 and XBP1 in a dose-dependent manner. HK-2 cells were transfected with specific KDM4C siRNA (A) and JMJD3 siRNA (B) or nonspecific scramble (Scr) siRNA at different doses for 48 h. Gene expression was assayed by quantitative RT-PCR using GAPDH as an internal control. Results are expressed as the mean ± SEM of three independent experiments. *p < 0.05 vs siRNA Scr.
